# Curcumin Loaded Nanocarriers with Varying Charges Augmented with Electroporation Designed for Colon Cancer Therapy

**DOI:** 10.3390/ijms23031377

**Published:** 2022-01-26

**Authors:** Julita Kulbacka, Kazimiera A. Wilk, Urszula Bazylińska, Magda Dubińska-Magiera, Stanisław Potoczek, Jolanta Saczko

**Affiliations:** 1 Department of Molecular and Cellular Biology, Faculty of Pharmacy, Wroclaw Medical University, Borowska 211A St., 50-556 Wroclaw, Poland; jolanta.saczko@umw.edu.pl; 2 Department of Engineering and Technology of Chemical Processes, Faculty of Chemistry, Wroclaw University of Technology, Wybrzeze Wyspianskiego 27, 50-370 Wroclaw, Poland; kazimiera.wilk@pwr.edu.pl; 3 Department of Physical and Theoretical Chemistry, Faculty of Chemistry, Wroclaw University of Technology, Wybrzeze Wyspianskiego 27, 50-370 Wroclaw, Poland; urszula.bazylinska@pwr.edu.pl; 4 Department of Animal Developmental Biology, Faculty of Biological Science, University of Wroclaw, Sienkiewicza 21, 50-335 Wroclaw, Poland; magda.dubinska-magiera@uwr.edu.pl; 5 Department of Hematology, Blood Neoplasms and Bone Marrow Transplantation, Wroclaw Medical University, Wybrzeże Pasteura 4, 50-367 Wrocław, Poland; stanislaw.potoczek@umw.edu.pl

**Keywords:** PLA nanocarriers, electroporation, surface charge, colon cancer, curcumin

## Abstract

(1) Background: The size and surface charge are the most significant parameters of nanocarriers that determine their efficiency and potential application. The poor cell uptake of encapsulated drugs is the main limitation in anticancer treatment. The well-defined properties of nanocarriers will enable to target specific tissue and deliver an active cargo. (2) Methods: In the current study, poly(D,L -lactide) (PLA) nanocarriers loaded with curcumin (CUR) and differing surface charge were evaluated for transport efficacy in combination with electroporation (EP) in dependence on the type of cells. The obtained CUR-loaded nanoparticles with diameters ranging from 195 to 334 nm (derived from dynamic light scattering (DLS)) were characterized by atomic force microscopy (AFM) (morphology and shape) and Doppler electrophoresis (*ζ*-potential) as well as UV-vis spectroscopy (CUR encapsulation efficiency (about 90%) and photobleaching rate). The drug delivery properties of the obtained PLA nanocarriers enhanced by electroporation were assessed in human colon cancer cells (LoVo), excitable normal rat muscle cells (L6), and free of voltage-gated ion channels cells (CHO-K1). CLSM studies, viability, and ROS release were performed to determine the biological effects of nanocarriers. (3) Results: The highest photodynamic activity indicated anionic nanocarriers (1a) stabilized by C_12_(COONa)_2_ surfactant. Nanocarriers were cytotoxic for LoVo cells and less cytotoxic for normal cells. ROS release increased in cancer cells with the increasing electric field intensity, irradiation, and time after EP. Muscle L6 cells were less sensitive to electric pulses. (4) Conclusions: EP stimulation for CUR-PLA nanocarriers transport was considered to improve the regulated and more effective delivery of nanosystems differing in surface charge.

## 1. Introduction

Colon cancer (CC) is still one of the most lethal cancers, with poor prognosis and late diagnosis in developing countries. The available data indicate that CC is the third most frequent malignant neoplasm in males and the second in females [1,2]. As a standard protocol, surgery, radio-, and chemotherapy are currently applied. Among the clinically approved chemotherapeutics are 5-FU, oxaliplatin, cisplatin, irinotecan, and their combinations [2,3,4]. Thus, the treatment optimization and new therapeutic approaches are intensively sought by researchers. Recently, natural compounds have been of interest as potential anticancer drugs, including colon cancer [5,6]. The available data highlight curcumin (diferuloylmethane, curcuma longa) as a promising natural chemotherapeutic that can suppress initiation, promotion, and progression of colon carcinogenesis [5,7,8,9,10,11]. Curcumin has been widely studied in terms of antioxidant, anti-inflammatory, and antimicrobial properties, as well as photosensitizing agents. Nevertheless, it still is not approved for clinical practice because of its low bioavailability and time-limited distribution in cancer cells [12,13]. This unfavorable feature can be related to curcumin’s instability at physiological pH and insolubility in water, which implicates slow uptake by cells and rapid metabolism inside cells [13]. Nanotechnology and electroporation can be engaged to meet these limitations and improve drug delivery. Both techniques can facilitate drug transport through cell membranes. Nanocarriers can protect the carried drug from the unfavorable external environment while maintaining their therapeutic usefulness, including chemical stability and photosensitivity, and control its release in targeted cells [14,15]. Curcumin was loaded effectively in microparticles [16], polysaccharides-based particles [17], and polymeric nanoparticles [16,18,19]. In turn, electroporation creates reorganization of lipids in cell membranes, which enables free diffusion of drug molecules [20,21,22,23]. The combination of both methods was also effectively applied in anticancer in vitro models for delivery of photosensitizers [24], big nanocarriers, or negatively charged nanosystems [25].

Here, we designed a study to determine (1) the anticancer effect of curcumin loaded in nanocarriers in three different cell lines, (2) a potential effect of various surface charges of used nanosystems, and (3) a stimuli effect of one electric pulse and irradiation.

## 2. Results and Discussion

**Scheme 1:**
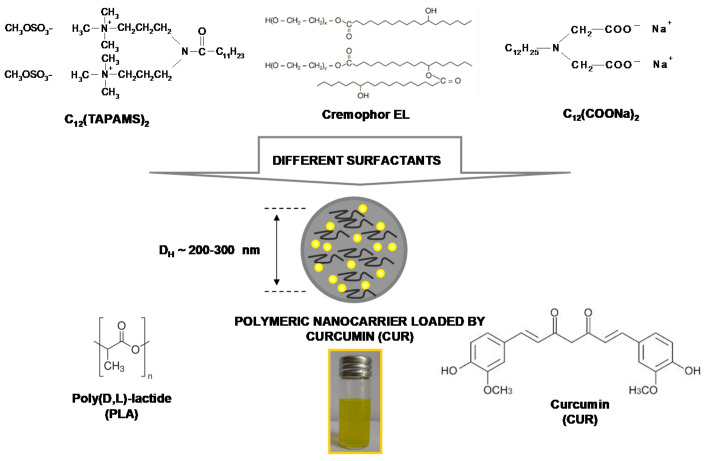
The representation of the nanosystems composition.

The available reports indicate poor bioavailability of curcumin [26,27]; its concentration is very low in body fluids and tissues [28]. It is caused mainly by poor absorption, a too-fast metabolism, and systemic elimination of curcumin [27]. Thus, novel strategies for its delivery are strongly wanted. The available studies revealed that curcumin could support standard chemotherapy [29] against various types of cancers, e.g., when used with cisplatin [30], docetaxel [31], or 5-fluorouracil [32]. This combination can be mainly implemented in the case of drug resistance, side effects, or specific selectivity to treatment [32]. It was also suggested that encapsulation and the usage of nanotechnology considerably enhance curcumin anticancer potential [26,33,34]. Here, we proposed an encapsulation method that was applied to fabricate colloidal-stable, resistant to chemical degradation, and biocompatible polymeric nanocarriers for effective curcumin (CUR) delivery to the target human colon cancer cells (LoVo). Our research is based on utilization of the nanoprecipitation method, where biocompatible polyester-origin polymer poly(D,L-lactide), PLA, and three surfactants with a different surface charge, i.e., cationic N,N-bis (3,3’-(trimethylammonio)-propyl) dodecanamide dimethylsulphate, C_12_(TAPAMS)_2_, anionic disodium N-dodecyliminodiacetate, C_12_(COONa)_2_ or non-anionic emulsifier-polyethoxylated castor oil, Cremophor EL, were applied to enhance the NPs stabilization (Scheme 1).

**Figure 1:**
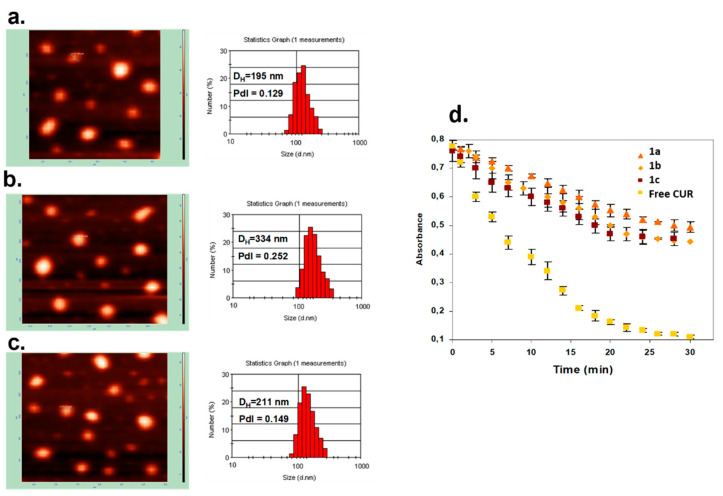
Characteristics of CUR-loaded nanocarriers. AFM images and DLS size distribution graphs of CUR-loaded nanocarriers stabilized by C_12_(TAPAMS)_2_ (**a**), C_12_(COONa)_2_ (**b**), and Cremophor EL (**c**); photobleaching of 4 μM encapsulated and free CUR in aqueous solution during irradiation with visible *light* (100 mW/cm²) measured by UV-Vis absorption spectra (**d**). Points present the difference in the absorption at λmax = 440 nm for loaded nanocarriers and λmax = 430 nm for the native form of photosensitizer as a function of time.

The physicochemical characteristics of the obtained NPs upon their size (hydrodynamic diameter, D_H_), surface charged expressed as ζ-potential, encapsulation efficiency (EE%), and CUR concentration are presented in Table 1. The values of the ζ-potential oscillated from −81mV (C_12_(COONa)_2_) through −9mV (Cremophor EL) to +42 mV (C_12_(TAPAMS)_2_), proving the colloidal stability as well as the influence of the applied surfactant type on the final NC surface charge. A similar impact was observed in the nanocarrier diameters, where we identified average sizes from 195 nm for the CUR-loaded NPs stabilized by C_12_(TAPAMS)_2_ to 334 nm for those containing C_12_(COONa)_2_. The narrow particle size distribution (PdI < 0.3) was detected in all cases. The size distribution graphs by number and by intensity were shown in Figure 1 and Figure S2, respectively. These results were supported by AFM morphology imaging, where the NPs spherical shape without any nanoparticle aggregation was detected (Figure 1). The high encapsulation efficiency (EE > 92%) proved the NC had a good capacity for CUR encapsulation. The effective photosensitiser encapsulation was also proved by almost equal zeta potential of empty and loaded nanocarriers (with a slightly smaller D_H_), excluding CUR adsorption on the NP surface [35].

The low photobleaching rate of the loading nanocarriers (Figure 1d) compared to free CUR molecules revealed nearly four times higher resistance of the encapsulated photosensitizer photodegradation in physiological fluids. The above features make the obtained nanocarriers promising candidates for effective drug delivery systems in photodynamic therapy (PDT) for maximum tumor damage via photoactivation [36]. However, different surface charges can have a crucial impact on the final cellular internalization and photodynamic efficiency.

**Figure 2:**
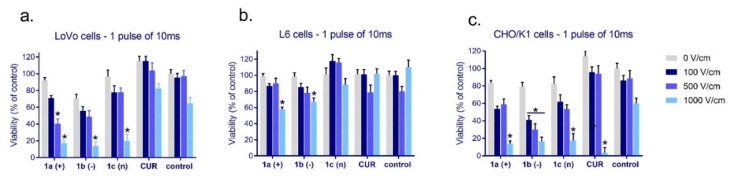
The cytotoxicity of free curcumin and curcumin ([CUR] = 4 μM) loaded in nanosystems evaluated after 24 h in (**a**) colon cancer cells (LoVo); (**b**) skeletal muscle cells (L6); and (**c**) hamster ovarian fibroblasts (CHO/K1). * *p* < 0.05.

The results from the evaluation of cellular viability in cancer (LoVo) and normal cells (L6 and CHO-K1) exposed to free and encapsulated curcumin combined with electroporation are presented in Figure 2. The least sensitive to curcumin alone, in nanosystems, or in combination with electroporation were muscle cells (L6, Figure 2b). The highest significant effect on cellular viability was observed when cells were exposed to 1000 V/cm with cationic and anionic CUR-nanosystems. In the case of colon cancer cells and normal fibroblasts, 500 and 1000 V/cm induced significant viability decrease in combination with all CUR-nanosystems (Figure 2a,c), and normal cells 1000 V/cm with free curcumin (Figure 2c). One electric pulse of 500 V/cm intensity provoked colon cancer cell viability decrease below 40% when cationic nanosystems were used and a 70% decrease in CHO-K1 cells (EP model) when anionic nanosystems were used with one pulse. The highest intensity of electric pulse induced a similar cytotoxic effect.

**Figure 3:**
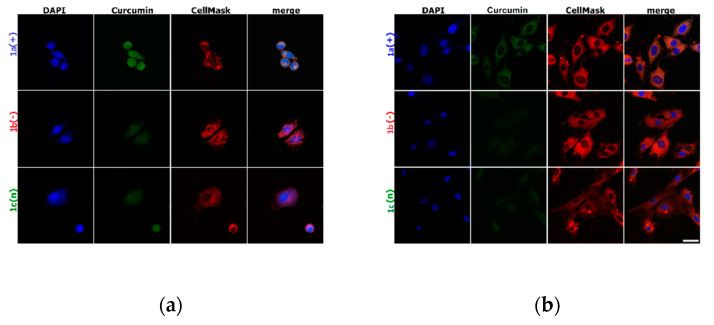

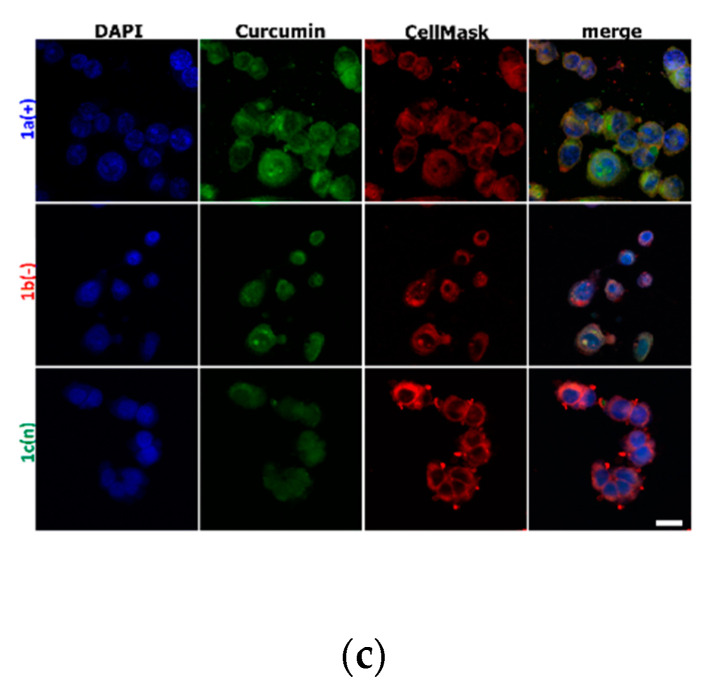
Cells incubated with encapsulated curcumin and stained for nuclei (DAPI) and cell membrane (CellMask DeepRed) visualization: (**a**) hamster ovarian fibroblasts (CHO-K1 cells); (**b**) normal rat skeletal muscle cells (L6 cells); (**c**) colon adenocarcinoma cells ( LoVo cells). The white scale bar corresponds to 10 µm. CUR concentration was 4 μM.

**Figure 4:**
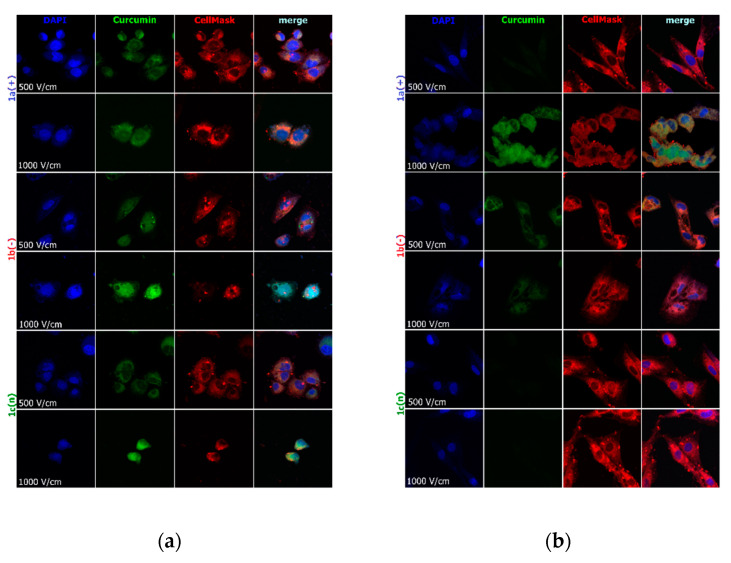

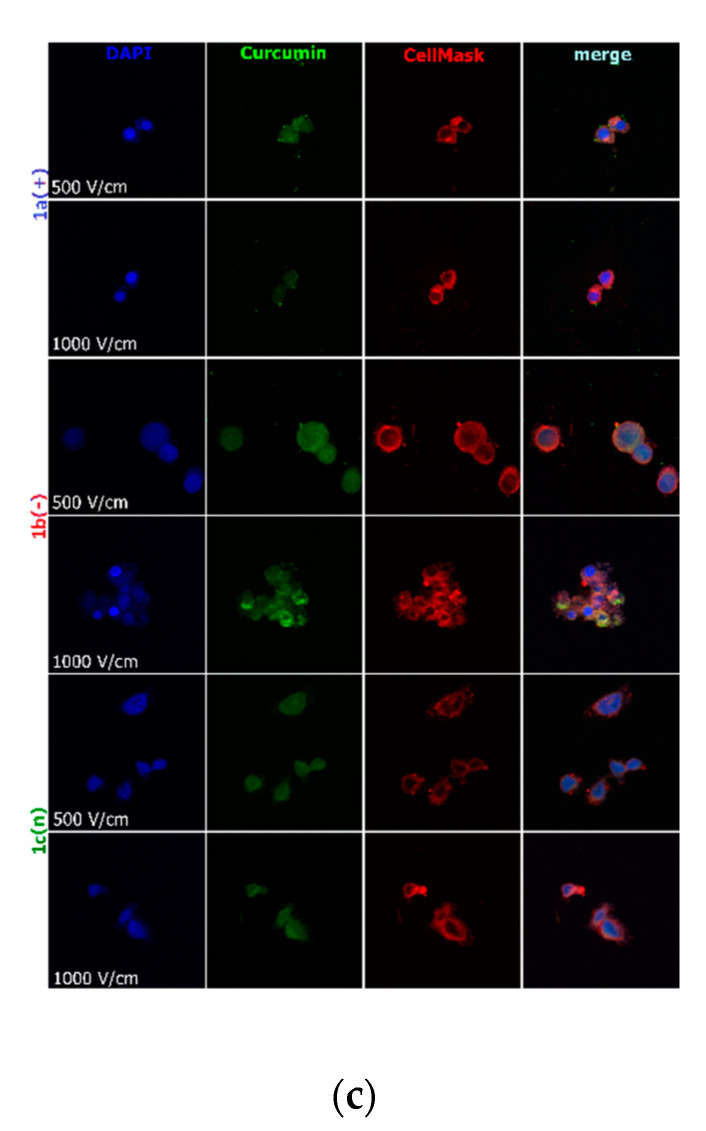
Cells electroporated (500 and 1000 V/cm) with encapsulated curcumin and stained for nuclei (DAPI) and cell membrane (CellMask DeepRed) visualization: (**a**) hamster ovarian fibroblasts (CHO-K1 cells); (**b**) normal rat skeletal muscle cells (L6 cells); (**c**) colon adenocarcinoma cells (LoVo cells). CUR concentration was 4 μM.

The imaging presented in Figure 3, Figure 4, and Figure S1 (Supplementary Material) represents the cell membrane morphology and curcumin distribution loaded in nanocarriers. Figure S1 shows the results obtained for control cells and cells exposed to 10 ms pulse. There was proved that one 10 ms electric pulse caused significant cell membrane reorganization, particularly in LoVo cells, hamster fibroblasts, and muscle L6 cells were almost intact. The intracellular distribution of variously charged CUR-nanosystems is demonstrated in Figure 3. As we can observe, the most intense fluorescent signal was observed for LoVo cells, in particular for 1a(+) nanosystems. Similarly, CHO-K1 cells also had the most effective uptake of this type of nanocarriers. The weakest fluorescent signal was detected for the negatively charged system 1b(−),particularly in normal cells.

Figure 4 visualizes the intracellular distribution of curcumin in nanoparticles delivered with the support of 10 ms electric pulse of the various electric field intensity to normal and cancer cells. CellMask DeepRed membrane and nuclei marker were used simultaneously to observe the effect on lipid membranes and nuclei. In the case of CHO-K1 and LoVo cells, cell shrinkage was noted with the increasing electric pulse intensity (Figure 4a,b). L6 cells were less sensitive to electric pulse, and no significant changes were observed. Only 1000 V/cm intensified the accumulation of the fluorescent signal derived from lipid membranes. The most efficient curcumin distribution was detected after encapsulation in 1b(−) nanosystems combined with 10 ms pulse of 1000 V/cm intensity in LoVo and CHO-K1 cells. Additionally, 500 V/cm was influential in the transport of all nanosystems. The weakest response for electroporation and nanosystems delivery revealed L6 cells, which are muscle cells. Our previous study also demonstrated the most efficient curcumin delivery occurred when electric field intensities higher than 600 V/cm were used in melanoma and skin cells [37].

The available study clearly indicates that curcumin encapsulation is promising against colon cancer [34,38,39]. The other research also reveals that electroporation may increase curcumin efficacy [40,41,42]. Yang et al. showed increased Cur stability and activity when loaded in micelles. The authors demonstrated a promising anticancer effect of Cur in MPEG-P(CL-co-TMC) micelles in CT26 colon carcinoma [38]. In the other study, biodegradable monomethoxy poly(ethylene glycol)poly(lactide) copolymer (MPEG-PLA) micelles were also effectively used to deliver curcumin to colon cancer cells [39]. There were also effectively used vehicles based on sodium alginate (micro- and macro-particles, uncoated, coated with chitosan or gelatin), where gelatin-based microparticles revealed the highest potential against LoVo cells [43].

**Figure 5:**
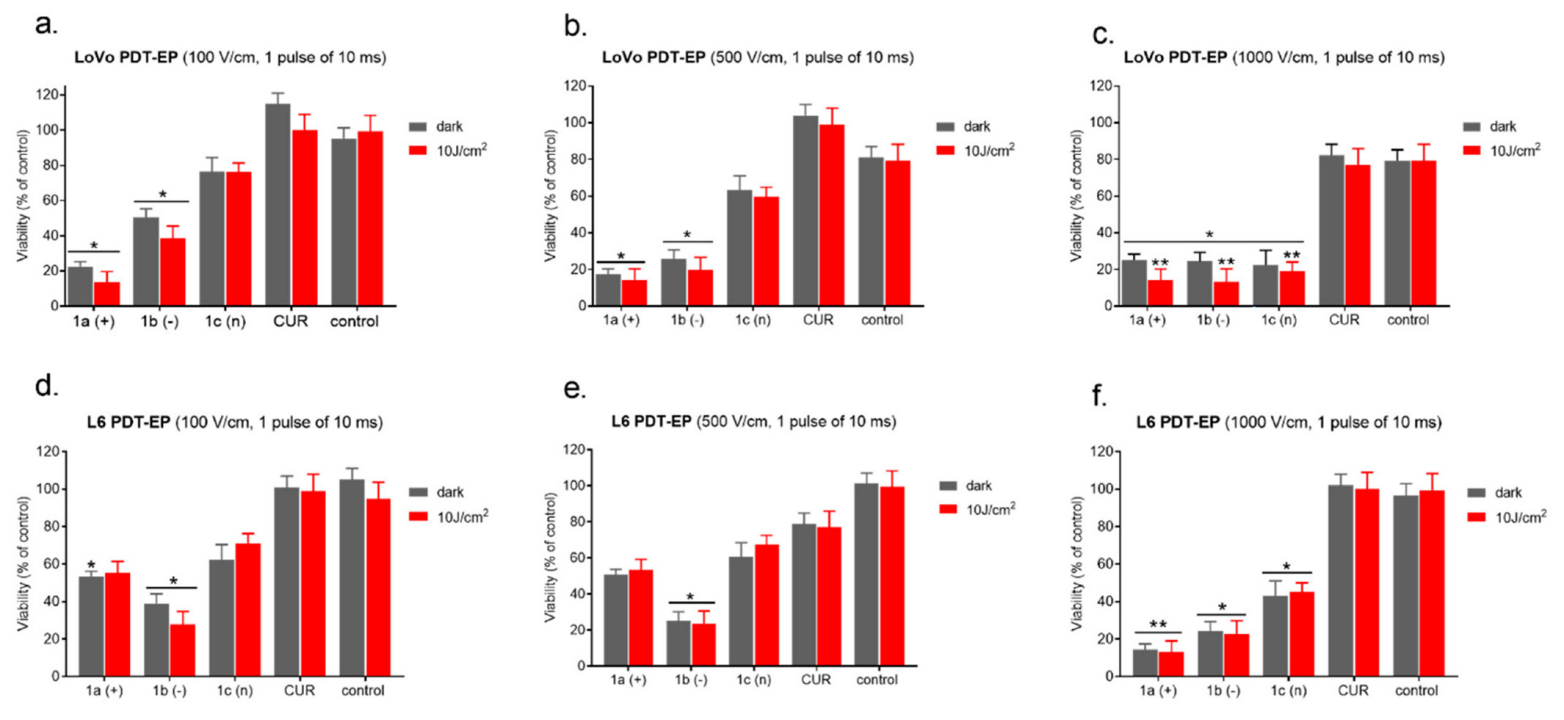
Photodynamic reaction after 10 min combined with EP in LoVo cells ((**a**–**c**)—upper panel) and normal L6 cells ((**d**–**f**)—bottom panel). CUR concentration was 4 μM. * p ≤ 0.05, ** *p* ≤ 0.01.

The evaluation of photocytotoxic effect enhanced by electroporation was presented in Figure 5a–f. Our results show that encapsulated Cur was significantly more cytotoxic than in free form (Figure 5). This effect was more evident in the case of colon cancer cells (LoVo—Figure 5a–c) than normal cells (L6—Figure 5d–f). Additionally, one 10 ms electric pulse of various electric field intensity was used to enhance nanosystems delivery, and then the irradiation (10 J/cm^2^) was proceeded.

**Figure 6:**
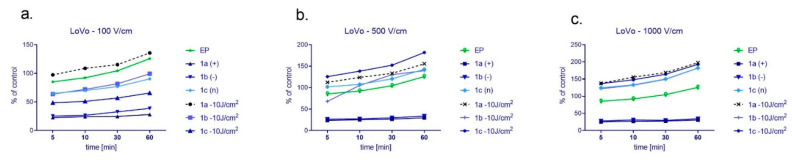
The level of ROS in colon cancer LoVo cells after cyto- and photocytotoxic reaction supported by electroporation using the following electric field intensity (**a**) 100 V/cm, (**b**) 500 V/cm, and (**c**) 1000 V/cm. Results were normalized and presented as a percentage of untreated control cells. CUR concentration was 4 μM.

**Figure 7:**
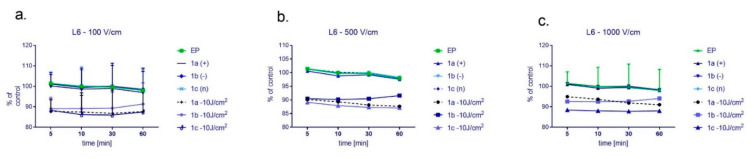
The level of ROS in normal L6 cells after cyto- and photocytotoxic reaction supported by electroporation using the following electric field intensity (**a**) 100 V/cm, (**b**) 500 V/cm, and (**c**) 1000 V/cm. Results were normalized and presented as a percentage of untreated control cells. CUR concentration was 4 μM.

Treatment of cancer (LoVo) and normal cells (L6) with nanoparticles of various charges induced an increase of reactive oxygen species (ROS) which was presented in Figure 6 (LoVo) and Figure 7 (L6). There was used DCF fluorescence assay to detect the level of ROS up to 60min post-irradiation. In the case of colon cancer cells (Figure 6), we could observe the rising ROS level in the time of detection and proportionally with the increasing EP intensity. The highest efficacy occurred after the exposure to 1a nanosystem and irradiation electroporated with 100 or 1000 V/cm. When cells were electroporated with 500 V/cm, then 1c nanosystem stimulated the highest ROS level. In general, when nanosystems were electroporated with 500 or 1000 V/cm and irradiated, reactive oxygen production was higher than in the other samples.

Normal cells (L6) revealed a completely different ROS release when exposed to the combined treatment (Figure 7). The highest ROS level was observed in cells exposed to electroporation alone or combined with nanosystems not irradiated. However, these differences were not evident, and the data in the graphs are not presented from zero, only from 80% of the value to show the differences in the measurements. Otherwise, ROS plots would be overlapped. It was also noted that ROS level is lower than in the case of colon cancer cells and is not increasing in the function of time.

The available studies demonstrate various effects on encapsulated curcumin and ROS release, e.g., apoptosis triggering [44]. It was also observed that immobilized curcumin in complex particles based on different polysaccharides expressed antioxidant activity against UV [17]. Curcumin incorporated in CAG nanocomposites (graphene-based) provoked a time-dependent increase in ROS with decreasing GSH and SOD levels in colon cancer cells (HT-29 and SW-948) [45]. However, in the other study, TPGS/CCM nanoparticles containing curcumin reduced the intracellular concentration of ROS, but stimulated apoptosis and slowed cell migration in HT-29 cells [46]. Similarly to our study, Yang et al. encapsulated curcumin in polymeric micelles for colon cancer cells (CT26 cells) treatment. Polymeric micelles enabled effective drug delivery and apoptosis in cancer cells [38]. Umerska et al. used polymeric nanoparticles based on two various polymers (PLGA and ERL) to increase oral or parenteral bioavailability of curcumin [19]. Our previous study revealed a promising application of electroporation for enhanced curcumin effect in glioma cells [47] and PDT with curcumin in melanoma cells [48], also combined with electroporation [37]. It was proved that irradiation of curcumin caused its degradation to vanillin, feruloylmethane, and ferulic acid. The combined protocols where EP and PDT was used caused selective genotoxicity towards malignant cells [37]. In turn, Weżgowiec et al. studied the effect of curcumin loaded in microparticles and macroparticles against human colon cancer cells (LoVo). It was shown that smaller nanosystems more efficiently delivered curcumin and intensified its anticancer effect [43]. In general, curcumin characterizes unsatisfactory biodistribution and tends to degrade; thus, new multifunctional nanosystems are highly required. The encapsulation process of curcumin causes its better intracellular distribution, targeting rate, and increased cytotoxic and anticancer effects [49]. In this study, we proposed novel multifunctional nanosystems with possible oral (anionic), transdermal (neutral), or injectable applications (cationic). Additionally, it was proved that one electric pulse can support the delivery of encapsulated curcumin in the targeted place, and if followed by irradiation, efficiently eliminate cancer cells.

## 3. Materials and Methods

### 3.1. Chemicals

All the reagents were purchased from Sigma Aldrich (Poznań, Poland) and used as received. Curcumin (Sigma-Aldrich, Poznań, Poland) from *Curcuma longa* was used as a photosensitizer. Poly(D,L-lactide) (PLA, *M*_W_~90,000–120,000) was employed as biocompatible polymer. Cremophor EL (polyethoxylated castor oil) was applied as a nonionic surfactant. Ionic surfactants: cationic N,N-Bis(3,30-(trimethylammonio)propyl)dodecanamide dimethylsulfate, C_12_(TAPMS)_2_, and anionic disodiumN-dodecyliminodiacetate C_12_(COONa)_2_ were synthesized according to the method described in our previous studies [50]. Water used in all the experiments was doubly distilled and purified employing a Millipore (Bedford, MA, USA) Milli-Q purification system.

### 3.2. Nanocarriers Preparation—Physicochemical Characterization of Nanoparticles

The poly(D,L-lactide) (PLA) nanocarriers/nanoparticles (NPs) were fabricated by the nanoprecipitation method—also called solvent displacement or interfacial deposition—corresponds as follows: for NPs preparation, the organic solution consisted of the PLA at a concentration (5 mg/mL) and curcumin (CUR) (0.5 mg/mL) dissolved in acetone. A constant volume (1 mL) of this organic phase was added dropwise under vigorous magnetic stirring to 5 mL of an aqueous solution containing a different type of surface active agent, i.e., dicephalic-type surfactants: cationic N,N-bis(3,3′-(trimethylammonio)-propyl)dodecanamide dimethylsulphate, C_12_(TAPAMS)_2_ and anionic disodium N-dodecyliminodiacetate, C_12_(COONa)_2_ or non-anionic emulsifier—polyethoxylated castor oil, Cremophor EL [51], at a concentration of 0.1 mg/mL. After 1 h of magnetic stirring, the organic solvent was evaporated at reduced pressure in a rotary evaporator (Büchi Rotavapor R-200), and NPs were collected overnight. In the next step, the suspension was separated via the nanofiltration process (cellulose nitrate membranes of pore size 500 nm) before size, morphology, ζ-potential, and photobleaching measurements. The representation of the nanosystems composition is shown in Scheme 1.

### 3.3. Evaluation of the NPsphysiochemical Parameters

The stability and particle charge of the obtained nanocarriers were evaluated by ζ-potential measurements using microelectrophoretic method (Malvern Zetasizer Nano ZS apparatus). All the measurements were performed at 25 °C. Each value was obtained as an average of three subsequent runs of the instrument with at least 20 measurements. The size (hydrodynamic diameter, D_H_) of the nanocarriers was determined by the dynamic light scattering technique—DLS (ZetaSizer Nano ZS, Malvern Instruments, Malvern, UK). All the measurements were performed at 25 °C with a detection angle of 173° in optically homogeneous square polystyrene cells. Each value was obtained as an average of three runs with at least ten measurements. The DTS (Nano) program was applied for data evaluation.

### 3.4. AFM Evaluation of Nanosystems Morphology

The morphology of the obtained drug delivery systems was examined by atomic force microscopy (AFM). Imaging was carried out using the ultra-low-amplitude tapping mode on a VeecoNanoScope Dimension V AFM with an RT ESP Veeco tube scanner. Before observations, the NPs were allowed to adsorb on a freshly cleaved mica surface for 12 h by dipping it in the suspension. Then, the excess of the substrate was removed by rinsing the mica plates in double-distilled water for 1 min and drying at room temperature.

### 3.5. Curcumin Encapsulation Efficiency

UV-vis spectroscopy (Metertec SP8001 spectrophotometer) was applied to determine the CUR encapsulation efficiency (EE%) and the photobleaching rate of the loaded or free photosensitizer. The UV absorbance measurements were performed using a spectrophotometer with 1 cm pathlength thermostated quartz cell, while the dye concentration was calculated using the calibration plot. All the measurements were performed in triplicate.

### 3.6. Cell Cultures

The studies were performed on human colon adenocarcinoma (LoVo) cells, hamster ovarian fibroblastoid (CHO-K1) cells, and rat myoblasts (L6). All cell lines were purchased from the ATCC^®^ (LGC Standards, Teddington, UK). CHO-K1 cells can be applied as a model for transport studies with the pulsed electric field due to deficient expression of endogenous ion channels [52]. This cell line was selected to model a study involving drug transport. LoVo cells were derived from human colon adenocarcinoma of type C by Dukes, and L6 cells originate from rat skeletal muscle cells. Ovarian fibroblasts were maintained in Ham’s F-12K (Kaighn’s modification) Medium (Gibco, Waltham, MA, USA), colon adenocarcinoma cell line was grown in Ham F-12 medium (Gibco, Waltham, MA, USA), and L6 cells were grown in Dulbecco’s Modified Eagle’s Medium (DMEM, Sigma-Aldrich, St. Louis, MI, USA). All culture media were supplemented with 10% fetal bovine serum (FBS, HyClone, Logan, UT, USA), 100 IU/mL penicillin, and 100 IU/mL streptomycin (Sigma-Aldrich, St. Louis, MI, USA). Cells were cultured in plastic flasks 25 or 75 cm^2^ (Nunc, Roskilde, Denmark), and stored at 37 °C and 5% CO_2_ in an incubator (SteriCult, ThremoScientific, Alab, Poland). The cells were detached for experiments by trypsinization (0.25% Trypsin-EDTA; Sigma-Aldrich, St. Louis, MI, USA) and neutralized by a sterile Dulbecco’s phosphate-buffered saline (DPBS, Sigma-Aldrich, St. Louis, MI, USA) for the experiments. The medium renewal was accomplished 2–3 times per week.

### 3.7. Electropermeabilization Protocol

Electropermeabilization (EP), alone and with free or encapsulated curcumin, was performed using ECM 830 Square Wave Electroporation System (BTX Harvard Apparatus, purchased from Syngen Biotech, Poland). After trypsinization and centrifugation (5 min, 1000 rpm), cells were counted and ((cells density) = 5 × 10^5^/mL) resuspended in 200 µL of SKM EP buffer with low electrical conductivity 0.14 S/m (10 mM KH_2_PO_4_/K_2_HPO_4_, 1 mM MgCl_2_, 250 mM sucrose, pH 7.4) [53]. In the case of nanosystems 1a, 1b, or 1c or CUR incubation, cells were suspended in EP buffer with CUR loaded in nanosystems or free curcumin where (CUR) = 4 μM. The cell suspension was pulsed in a cuvette with two aluminum plate electrodes with electrical field strength E(appl) = 100, 500, and 1000 V/cm using one pulse of 10 ms duration. A rectangular electrical pulse was delivered by electroporator ECM 830 (BTX, Syngen Biotech Poland). After pulsation, before further experiments, the cells were left for 10 min at 37 °C, centrifuged (1000× *g*), resuspended in cell culture medium, and designed for viability, CLSM, or ROS assay.

### 3.8. Confocal Laser Scanning Microscopy for Nanocarriers Bioimaging

CLSM method was applied for the intracellular curcumin distribution and cell membrane condition in cancer (LoVo) and normal cells (L6 and CHO-K1). Cells were seeded on microscopic cover slides placed in Petri dishes (35 mm, Nunc, Poland) for 24 h, then cells were exposed to nanosystems (at 4 μM curcumin concentration), or electroporated (using Petri Dish 35 mm electrode—BTX, Syngen, Poland) with UCs CUR-based nanosystems and incubated for 24 h. In slides exposed to electroporation, the following steps were the same as in the previous *Electropermeabilization protocol*. After the incubation, the culture medium was removed, and the cover glasses were washed with PBS and fixed for 10 min in 4% paraformaldehyde (PFA) and washed with PBS. Slides were mounted in fluorescence mounting medium (DAKO), containing DAPI (4,6-diamidino-2-phenylindole) for nuclei staining. The preparations were stored in the darkness in 4 °C. FluoView FV1000 confocal laser scanning microscope (Olympus, Shinjuku-ku, Japan) was used for imaging.

### 3.9. Cell Viability Assay

The cell viability was performed using the MTT cell proliferation assay (Sigma Aldrich, St. Louis, MO, USA). First, cells (LoVo, L6, and CHO/K1) were subjected to EP (see Section 3.7) alone, or with free curcumin, or curcumin loaded in nanosystems (1a (+), 1b (−) and 1c (n)) in the final concentration 4 µM. After that, cells were incubated for 10 min at 37 °C and 5% CO_2_ for membrane resealing. Then, cells were resuspended onto 96-well plates (Sarstead, Nümbrecht, Germany) at a density of 3 × 10^4^ cells. Additionally, the photodynamic potential of free and encapsulated curcumin was verified, and cells were irradiated by 10 J/cm^2^ light dose using fiber illuminator (OPTEL, Opole, Poland) with polarized light (fluence rate at the level of the cell monolayer: 10 mW/cm^2^) and a red filter (λmax = 632.8 nm). Then, viability was performed after 24 h according to the manufacturer protocol and our previous study [54]. The absorbance was measured at the wavelength of 570 nm using a microplate reader (EnSpire, Perkin Elmer, Waltham, MA, USA). The cell viability measured for each group was expressed as a percentage of control untreated cells. All experiments were performed in triplicate.

### 3.10. Intracellular ROS Production

For the ROS production, after electroporation and electroporation combined with photodynamic therapy, cells were cultured on black 96-well plates with the flat transparent bottom (Perkin Elmer, Poland) in concentration 3 × 10^4^ per well. DCF protocol was measured after 5, 10, 30, and 60 min post-irradiation by 10 J/cm^2^, and then after 10 min. Reactive oxygen production after photodynamic reaction (PDR) was determined using DCF (2,7-dichlorofluorescein) assay (Life Technologies, Carlsbad, CA, USA). This DCF assay is based on the application of fluorescent properties of 6-carboxy-2,7-dichlorodihydrofluorescein diacetate 2,7-dichlorofluorescein (carboxy-H_2_DCFDA). For experimentations, the concentrated stock solution of carboxy-H_2_DCFDA (50 µg/mL in sterile DMSO) was kept at RT in the dark conditions and was subsequently properly diluted, following the manufacturer’s protocol, in cell culture medium without FBS. Then, the incubation medium was washed out from the cells with PBS containing 6 mM glucose, and the DCF reagent was added to the cell culture to achieve a final concentration of 10 µM and was left in darkness for 30 min, with incubation at 37 °C. After this time, the fluorescence was measured, where the excitation wavelength of 495 nm and emission wavelength of 530 nm was used. ROS level was detected by a multiwell scanning spectrophotometer (EnSpire Perkin Elmer, Poland).

### 3.11. Statistical Analysis

All the data are presented as mean values ± SD calculated from a minimum of three independent experiments. The two-way Anova test was used to check the significance level between independent variables. The significance level was set to *p <* 0.005 or *p <* 0.05.

## 4. Conclusions

Summarizing, we can state that electroporation with only one pulse enhances free and encapsulated curcumin’s delivery. The highest photodynamic potential indicated anionic nanocarriers (1a) stabilized by C_12_(COONa)_2_ surfactant (cytotoxic in LoVo, low toxicity in normal cells). Reactive oxygen species release increased in cancer cells with the increasing electric field intensity and time after electroporation. Normal muscle L6 cells are an excitable type of cells and thus were less sensitive to electric pulses. The microscopic evaluation study revealed that curcumin was the most efficiently delivered by cationic nanoparticles in CHO-K1 and LoVo cells. Curcumin in nanosystems revealed good biodistribution and indicated anticancer properties against the human colon cancer model in vitro. Thus, the application of nanosystems with various surface charge can be considered for future in vivo applications.

## Figures and Tables

**Table 1:** Characteristics of CUR-loaded and empty polymeric nanocarriers.

System	Composition	C_CUR_ [μM]	EE [%]	D_H_ [nm]	PdI	ζ [mV]
1a	C_12_(TAPAMS)^2^/PLA/water	77.9	92	195 ± 2	0.129 ± 0.01	+42 ± 3
1b	C_12_(COONa)_2_/PLA/water	80.9	95	334 ± 7	0.252 ± 0.02	−81 ± 6
1c	Cremophor EL/PLA/water	88.2	93	211 ± 4	0.149 ± 0.01	−9 ± 1
2a	C_12_(TAPAMS)_2_/PLA/water	EMPTY		186 ± 2	0.129 ± 0.01	+41 ± 3
2b	C_12_(COONa)_2_/PLA/water	EMPTY		310 ± 6	0.232 ± 0.02	−80 ± 6
2c	Cremophor EL/PLA/water	EMPTY		207 ± 3	0.147 ± 0.01	−9 ± 1

D_H_: hydrodynamic diameter (Z-Ave), PdI: polydispersity index, ζ: zeta potential, EE: encapsulation efficiency.

## Data Availability

Not applicable.

## Supplementary Materials

The following supporting information can be downloaded at: https://www.mdpi.com/article/10.3390/ijms23031377/s1.
