# Supplementary material for: Curcumin Loaded Nanocarriers with Varying Charges Augmented with Electroporation Designed for Colon Cancer Therapy
Source: Int J Mol Sci. 2022 Jan 26;23(3):1377. doi: 10.3390/ijms23031377 (PMC8836164; doi:10.3390/ijms23031377)
Supplement: Supplementary file 1 [file ijms-23-01377-s001.zip › ijms-1555671-Supplementary.pdf]

ELECTRONIC SUPPLEMENTARY INFORMATION

Curcumin loaded nanocarriers about variable charge augmented with electroporation designed for colon cancer therapy

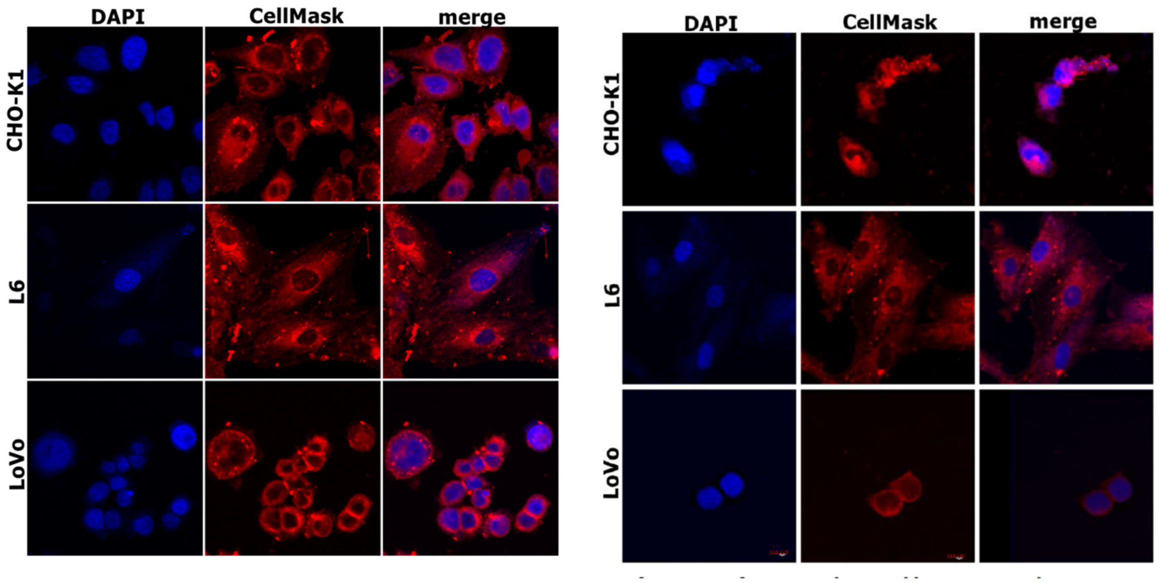

(a) (b)

Figure S1 Cells with stained nuclei (DAPI) and cell membrane marker CellMask: (**a**) control cells; (**b**) cells electroporated with 1000 V/cm.

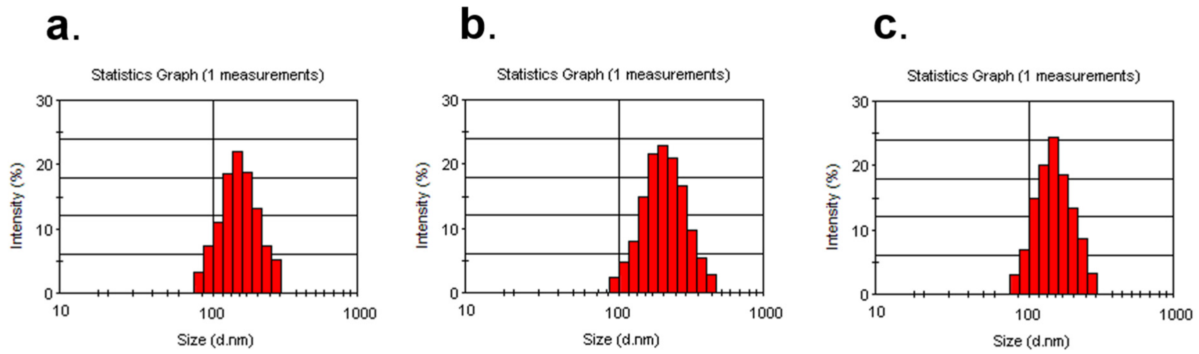

Figure S2. DLS size distribution graphs (by intensity) of CUR-loaded nanocarriersstabilized by $C_{12}(TAPAMS)_2$ (**a**), $C_{12}(COONa)_2$ (**b**), and Cremophor EL (**c**);
